# Expertenempfehlung: Therapie nichtgehfähiger Patienten mit Muskeldystrophie Duchenne

**DOI:** 10.1007/s00115-020-01019-3

**Published:** 2020-11-19

**Authors:** Guenther Bernert, Andreas Hahn, Cornelia Köhler, Sascha Meyer, Ulrike Schara, Kurt Schlachter, Regina Trollmann, Maggie C. Walter

**Affiliations:** 1 Sozialmedizinisches Zentrum Süd, Kaiser-Franz-Josef-Spital mit Gottfried von Preyer’schem Kinderspital, Wien, Österreich; 2 Abteilung Kinderneurologie, Sozialpädiatrie und Epileptologie, Zentrum Kinderheilkunde, Justus-Liebig-Universität, Gießen, Deutschland; 3 Abteilung Neuropädiatrie, Sozialpädiatrie, Klinik für Kinder- und Jugendmedizin, Ruhr-Universität Bochum, Bochum, Deutschland; 4 Sektion Neuropädiatrie, Klinik für Allgemeine Pädiatrie und Neonatologie, Universitätsklinikum des Saarlandes, Homburg, Deutschland; 5 Abteilung für Neuropädiatrie, Zentrum für neuromuskuläre Erkrankungen des Kindes- und Jugendalters, Universitätsklinikum Essen, Universität Duisburg-Essen, Essen, Deutschland; 6 Abteilung Kinder- und Jugendheilkunde, Landeskrankenhaus (LKH) Bregenz, Bregenz, Österreich; 7 Abteilung Neuropädiatrie und Sozialpädiatrisches Zentrum, Kinder- und Jugendklinik am Universitätsklinikum, Friedrich-Alexander-Universität Erlangen-Nürnberg, Erlangen, Deutschland; 8 Friedrich-Baur-Institut, Neurologische Klinik und Poliklinik, Klinikum der Ludwig-Maximilians-Universität München, Ziemssenstr. 1, 80336 München, Deutschland

**Keywords:** Muskeldystrophie Duchenne, Therapie, Glukokortikoide, Ataluren, Exon-Skipping, Idebenon, Duchenne muscular dystrophy, Pharmacological therapy, Glucocorticoids, Ataluren, Exon skipping, Idebenone

## Abstract

**Hintergrund:**

Die Muskeldystrophie Duchenne (DMD) ist die häufigste genetische neuromuskuläre Krankheit im Kindesalter, bei der es meist im Alter von 9 bis 11 Jahren zum Verlust der Gehfähigkeit kommt.

**Ziel der Arbeit und Material und Methoden:**

Auf der Grundlage aktueller Leitlinien und Studien erarbeiteten neuropädiatrische und neurologische Experten im Rahmen eines von der Firma PTC Therapeutics GmbH (Frankfurt am Main, Deutschland), die die Substanz Ataluren vertreibt, gesponserten Advisory Boards Empfehlungen zur Behandlung nichtgehfähiger Patienten mit DMD mit Schwerpunkt medikamentöse Therapien von Erwachsenen.

**Ergebnisse und Diskussion:**

Der Verlust der Gehfähigkeit wird in Studien sehr unterschiedlich definiert und bezieht sich u. a. auf die Rollstuhlpflicht, das selbständige Gehen ohne Hilfsmittel oder die maximale Gehstrecke. Grundlage der Therapie von Patienten mit DMD in jedem Krankheitsstadium sind supportive und symptomatische Maßnahmen, die in der Regel auch nach dem Verlust der Gehfähigkeit intensiv weitergeführt werden sollten. Zusätzlich stehen den Patienten medikamentöse Therapien mit dem Ziel der Modifikation des Krankheitsverlaufes zur Verfügung. Glukokortikoide bilden den Stützpfeiler der medikamentösen Therapie auch über den Verlust der Gehfähigkeit hinaus, dann meist in reduzierter Dosis. Für Patienten mit DMD aufgrund einer Nonsense-Mutation (nmDMD), ca. 13 % aller DMD-Patienten, steht Ataluren als potenziell dystrophinwiederherstellende, krankheitsmodifizierende Therapie zur Verfügung; klinische Daten aus dem STRIDE-Register zeigen eine verzögerte Krankheitsprogression auch nach Verlust der Gehfähigkeit. Zum Exon-Skipping liegen für erwachsene Patienten derzeit noch keine belastbaren Daten vor. Das Antioxidans Idebenon kommt bei nichtgehfähigen, jugendlichen Patienten ohne therapeutische Alternative, die nicht mit Glukokortikoiden behandelt werden können, infrage. Ataluren eignet sich zur kombinierten Behandlung mit Glukokortikoiden, eine Kombination von Idebenon und Glukokortikoiden wird derzeit in einer klinischen Studie überprüft. Eine Add-on-Therapie mit Idebenon zusätzlich zu Ataluren ist bei nichtgehfähigen nmDMD-Patienten zu erwägen. Bedingt durch die Tatsache, dass sich einige der diskutierten Therapieoptionen noch in der Phase der klinischen Prüfung befinden oder noch keine oder nur begrenzte Daten für ältere Patienten mit DMD vorliegen, handelt es sich um Expertenempfehlungen entsprechend der Evidenzklasse IV.

## Abstract (en)

**Background:**

Duchenne muscular dystrophy (DMD) is the most frequent genetic neuromuscular disease in childhood with loss of ambulation usually occurring around the age of 9–11 years.

**Objective, material and methods:**

Based on current guidelines and clinical trials, neuropediatric and neurological experts developed recommendations for the treatment of nonambulatory DMD patients focusing on drug treatment of adults. This advisory board was sponsored by PTC Therapeutics, the distributers of the substance ataluren.

**Results and conclusion:**

Loss of ambulation is heterogeneously defined across clinical trials. Among others, the need of a wheelchair, ambulation without mobility aids or maximum walking distance can be suitable parameters for assessment. Treatment of DMD patients at any stage of the disease is based on supportive and symptomatic measures, which should be continued after loss of ambulation. In addition, disease-modifying drugs are available for the treatment of DMD and glucocorticoids are the usual standard of care treatment even beyond the loss of ambulation. Ataluren, a potentially dystrophin restorative, disease-modifying treatment, has been approved for patients with DMD due to a nonsense mutation (nmDMD), which applies to approximately 13% of DMD patients and is usually combined with steroids. Clinical data from the STRIDE registry demonstrated a delayed disease progression even after loss of ambulation. Currently, no reliable data are available for exon skipping approaches in adult DMD patients. The antioxidant idebenone could be an option in nonambulant adolescent patients not treated with glucocorticoids and without other therapeutic options. A combination treatment of idebenone and glucocorticoids is currently being investigated in a clinical trial. Add-on treatment with idebenone in addition to ataluren may be considered for nonambulant nmDMD patients. Some of the discussed treatment options are still in clinical trials or there are not enough data for older DMD patients; therefore, these expert recommendations correspond to evidence class IV.

Die Muskeldystrophie Duchenne (DMD) ist die häufigste genetische neuromuskuläre Krankheit im Kindesalter. Sie ist durch eine zunehmende Verschlechterung motorischer Funktionen mit Verlust der Gehfähigkeit im Alter von 9 bis 11 Jahren gekennzeichnet. Der Verlust der Gehfähigkeit bedeutet für die Patienten und ihre Familien eine einschneidende Veränderung ihrer Lebenssituation. Insbesondere kommt es zu erheblichen Veränderungen des Spektrums der klinischen Problemfelder und neuen therapeutischen Herausforderungen.

## Hintergrund

Die DMD ist eine X‑chromosomal rezessive Erkrankung, die überwiegend Jungen betrifft. Die Inzidenz beträgt schätzungsweise 1:3600 bis 1:6000 der männlichen Neugeborenen [5]. Die Erkrankung wird durch Mutationen im Dystrophingen verursacht, die zu einem Mangel an funktionsfähigem Muskelstrukturprotein Dystrophin führen. Bei etwa 10–15 % der Patienten liegt der Erkrankung eine Nonsense-Mutation (nmDMD) zugrunde [30]. Der Dystrophinmangel geht mit einem vorzeitigen Untergang von Muskelzellen, einer zunehmenden Muskelschwäche und der Ausbildung von Kontrakturen einher.

Zu einem Verlust der Gehfähigkeit mit Rollstuhlabhängigkeit kommt es im Durchschnitt im Alter von 9,5 Jahren [15]. Gehverlust wird dabei in Studien sehr unterschiedlich definiert. Die Definitionen reichen von einer zeitweise oder dauerhaften Rollstuhlpflicht [27, 31, 33, 36] bis zur Unfähigkeit der Patienten, selbstständig ohne Stützen oder Hilfsmittel zu gehen [11]. Strengere Definitionen verwenden die maximal mögliche Gehstrecke als Kriterium für Nichtgehfähigkeit, beispielsweise weniger als 10 m oder 0 m innerhalb von 6 min [28, 32]. Einer aktuellen, allerdings weniger akzeptierten Definition zufolge besteht Nichtgehfähigkeit, wenn Patienten mehr als 30 s benötigen, um ohne Hilfe eine Distanz von 10 m zu überwinden oder innerhalb von 6 min eine Gehstrecke von weniger als 300 m erreichen [19].

Meilensteine der Krankheitsprogression bei nichtgehfähigen Patienten betreffen hauptsächlich die Funktion der oberen Extremitäten sowie die Lungen- und Herzfunktion [2, 3, 14]. Instrumente für die Beurteilung der motorischen Funktionen der oberen Extremitäten sind u. a. der Performance of Upper Limb (PUL), der MyoGrip und MyoPinch [32]. Daten bei Erwachsenen mit DMD liegen derzeit nicht vor. Für die Einschätzung der Lungenfunktion werden insbesondere die forcierte Vitalkapazität (FVC), aber auch der exspiratorische Spitzenfluss (PEF) herangezogen. Bei einer vorhergesagten FVC (FVC%p) <60 % ist die Indikation zur Atemtherapie gegeben, und bei einer FVC%p <50 % bzw. bei reduziertem Hustenstoß besteht in den meisten Fällen eine Indikation für atem- und hustenunterstützende Therapieformen sowie im Verlauf für eine nichtinvasive Beatmung [3]. Respiratorische Komplikationen treten in späteren Krankheitsstadien umso später auf, je länger zuvor die Gehfähigkeit aufrechterhalten werden konnte [18]. Wichtigste Parameter für die Beurteilung der Herzfunktion sind die echokardiographisch bestimmte linksventrikuläre Ejektionsfraktion (LVEF) und die Verkürzungsfraktion (SF). Eine überwachungspflichtige Kardiomyopathie im Rahmen einer DMD besteht bei einer LVEF <55 % oder einer SF <28 % sowie bei rascher Dynamik [35]. In Ergänzung der Echokardiographie hilft die Kardio-MRT vor kreislaufbelastenden operativen Eingriffen, z. B. wirbelsäulenaufrichtenden Operationen, das Ausmaß der Myokardfibrose besser einzuschätzen.

Die Therapie sowohl gehfähiger als auch nichtgehfähiger Patienten mit DMD ist interdisziplinär ausgerichtet. Überwiegend wird dies in interdisziplinären Muskelzentren oder sozialpädiatrischen Zentren realisiert. Die Lotsenfunktion nimmt dabei die Neuropädiatrie, nach der Transitionsphase die Neurologie, bevorzugt im Rahmen neuromuskulärer Zentren ein (https://www.dgm.org/medizin-forschung/neuromuskulaere-zentren-dgm). Ziele der interdisziplinären Therapie sind die Kontrolle oder Vermeidung sekundärer Komplikationen unter Einbeziehung der psychosozialen Situation. Weitere Therapieziele stellen die Verzögerung der Krankheitsprogression sowie eine Verbesserung der Lebenserwartung und Lebensqualität dar [3]. Mit der Etablierung eines interdisziplinären Therapiekonzepts und Einführung von Therapieoptionen wie chirurgische Wirbelsäulenstabilisation, Beatmungsverfahren, Glukokortikoidtherapie und kardiologische Betreuung hat sich die Lebenserwartung von Patienten mit DMD von 20 bis 30 Jahren auf jetzt 40 bis 50 Jahre verbessert [40].

Die vorliegenden Empfehlungen sind Teil eines von der Firma PTC Therapeutics GmbH (Frankfurt am Main, Deutschland), die die Substanz Ataluren vertreibt, unterstützten Meetings, ohne dass eine Einflussnahme der Firma auf die Diskussionen und Ergebnisse erfolgte. Teilnehmer waren Experten aus deutschsprachigen Ländern, die in größeren neuromuskulären Zentren tätig sind und über langjährige Erfahrung in der Betreuung einer größeren Zahl von Patienten mit DMD verfügen. Aufgrund der vielfältigen Forschungsansätze auf dem Gebiet der neuromuskulären Erkrankungen und der DMD im Besonderen erfolgte die Literaturrecherche als Scoping-Review. Die Empfehlungen wurden einstimmig verabschiedet. Bedingt durch die Tatsache, dass sich einige der diskutierten Therapieoptionen noch in der Phase der klinischen Prüfung befinden oder noch keine oder nur begrenzte Daten für ältere Patienten mit DMD vorliegen, handelt es sich um Expertenempfehlungen entsprechend der Evidenzklasse IV.

## Supportive Therapiemaßnahmen

Supportive und symptomatische Maßnahmen stellen in jedem Krankheitsstadium die Grundlage der Behandlung von DMD-Patienten dar und umfassen nichtmedikamentöse, medikamentöse und chirurgische Verfahren. Detaillierte, an die jeweilige Krankheitsphase adaptierte Empfehlungen sprechen die 2018 veröffentlichten Leitlinien von Birnkrant et al. zur Diagnose und Therapie der DMD aus [2, 3]. Diese werden nachfolgend im Hinblick auf nichtgehfähige Patienten zusammenfassend skizziert.

### Nichtmedikamentöse und chirurgische Therapien

Physiotherapie, zunehmend bezogen auf die obere Extremität und die Wirbelsäule, sowie Hilfsmittel wie Lagerungsschienen und Orthesen dienen auch nach Verlust der Gehfähigkeit der Vorbeugung zusätzlicher Funktionseinschränkungen durch Kontrakturen und Gelenkfehlstellungen. Insbesondere sollte auf eine Kontrakturprophylaxe mit physiotherapeutischen Maßnahmen Wert gelegt werden. Positiv auf die Prävention einer Skoliose wirkt sich aus, wenn Patienten regelmäßig, unterstützt durch Orthesen, Stehständer oder Rollstühle mit Aufstehhilfe zum Stehen angehalten werden; dies ist auch im Rahmen eines ganzheitlichen Schmerzmanagements ein wichtiger Baustein der Therapie. Wichtig ist die Frakturprävention und ggf. medikamentöse Osteoporosetherapie mit Kalzium, Vitamin D und ggf. Bisphosphonaten sowie eine regelmäßige DEXA-Kontrolle zur Graduierung einer Osteoporose. Auch kommen Weichteil- bzw. knöcherne Operationen im Bereich der unteren Extremitäten oder der Wirbelsäule bzw. Verlängerungsoperationen wie z. B. „Release“-Operationen von sekundär verkürzten Muskelgruppen in Betracht. Bei nicht mehr gehfähigen Patienten treten im Krankheitsverlauf zunehmend respiratorische und kardiale Komplikationen in den Vordergrund. Impfungen zur Pneumonieprophylaxe und Atemtherapie werden als sinnvoll erachtet [2]. Außerdem ist die Indikation zur Lungenvolumenrekrutierung sowie für atem- und hustenunterstützende Maßnahmen bis hin zur nichtinvasiven oder invasiven Beatmung frühzeitig zu prüfen. Bei ersten Hinweisen für eine Herzinsuffizienz ist eine pharmakologische Therapie, insbesondere die frühzeitige Gabe eines ACE-Hemmers indiziert.

Bei Übergewicht als Folge von Bewegungsmangel oder Steroidtherapie ist eine frühzeitige Ernährungsberatung angezeigt. Bei Untergewicht in Folge einer respiratorischen Verschlechterung oder aufgrund erschwerter Nahrungsaufnahme durch Dysphagie mit der Gefahr von Malnutrition sollte die Anlage einer PEG-Sonde in Erwägung gezogen werden, bei Osteoporose ggf. die Gabe von Kalzium und Substitution mit Vitamin D. Bei durch die Langzeitsteroidtherapie verursachtem Kleinwuchs kann in Einzelfällen eine Wachstumshormontherapie in Erwägung gezogen werden [5]. Bei Hypogonadismus sollte die Behandlung mit männlichem Sexualhormon gemeinsam mit den pädiatrischen Endokrinologen diskutiert werden, eine Substitution mit Testosteron kann hier hilfreich sein.

Zur Unterstützung psychischer und psychosozialer Belastungen und Beeinträchtigungen kommen psychotherapeutische Verfahren, ggf. auch eine medikamentöse Therapie infrage. Mit interdisziplinären Strukturen sollten soziale Interventionen einschließlich Bildungsmaßnahmen und Förderung des Schulbesuchs im Bedarfsfall angeregt werden. Die psychosoziale Unterstützung sollte die Familie des Patienten ebenfalls mit einbeziehen.

### Konsensus

Supportive und symptomatische Maßnahmen stellen die Grundlage der Therapie von Patienten mit DMD in jedem Krankheitsstadium dar. Therapiemaßnahmen wie z. B. Physiotherapie sollten nach Verlust der Gehfähigkeit weitergeführt werden. Bei nichtgehfähigen Patienten tritt zunehmend die Therapie respiratorischer und kardialer Komplikationen in den Vordergrund [15].

Ein besonderes Augenmerk sollte der psychischen und psychosozialen Situation der Patienten und ihrer Familien gelten.

## Medikamentöse Therapien

Zusätzliche medikamentöse Therapien haben insbesondere eine Modifikation des Krankheitsverlaufs im Blick und/oder zielen kausal auf die genetische Ursache der DMD ab. Tab. 1 zeigt eine Übersicht über Substanzen, die im Rahmen klinischer Studien untersucht wurden oder noch geprüft werden. Die Datenlage zur Wirksamkeit medikamentöser Therapien bei nichtgehfähigen DMD-Patienten ist begrenzt.

**Table 1:** 

Klasse	Wirksubstanz	Wirkmechanismus	Zulassung für DMD
Glukokortikoide	Deflazacort Prednison	Nicht vollständig geklärt, antiinflammatorische Wirkung	Ja (nur FDA), „off-label use“ weltweit
	*Vamorolon (synthetisches Glukokortikoid)*	*Nicht vollständig geklärt, antiinflammatorische Aktivität*	*Nein*
Mutationsspezifische Medikamente	Ataluren	„Read-through“ vorzeitiger Stopp-Codons	Ja (nur EMA)
	*Eteplirsen*	*Exon-51-Skipping*	*Ja (nur FDA)*
	*Golodirsen*	*Exon-53-Skipping*	*Ja (nur FDA)*
Koenzym-Q10-Analogon	*Idebenon*	*Antioxidans*	*Nein*

*DMD* Duchenne muscular dystrophy, *EMA* European Medicines Agency, *FDA* Food and Drug Administration

### Glukokortikoide

Glukokortikoide sind seit langem in der Therapie der DMD als Standard etabliert. In randomisierten Studien konnte eine Verzögerung von Muskelkraft- und Funktionsverlust einschließlich eines längeren Erhalts der Gehfähigkeit nachgewiesen werden [21]. Ihre Wirksamkeit beruht wahrscheinlich vor allem auf ihrer antiinflammatorischen Aktivität. Im Hinblick auf die Wirksamkeit bei nichtgehfähigen Patienten ist ein längerer Erhalt der Funktion der oberen Extremitäten, die verzögerte Entwicklung respiratorischer und kardialer Komplikationen sowie die Vermeidung von Skolioseoperationen von Bedeutung [3].

Die Gabe eines Glukokortikoids ist für jeden DMD-Patienten ab einem Alter von 5 Jahren zu erwägen, möglichst aber vor dem Stillstand der motorischen Entwicklung bzw. vor beginnenden Muskel- und Sehnenverkürzungen [3]. Sofern dem keine relevanten Nebenwirkungen entgegenstehen, sollte die Therapie zumindest in reduzierter Dosis nach Verlust der Gehfähigkeit fortgeführt werden. Ein Therapiebeginn erst nach dem Verlust der Gehfähigkeit ist ebenfalls möglich [40]. Zum Einsatz kommen überwiegend Prednison oder Deflazacort. Für Valmorolon als synthetisches Glukokortikoid liegen derzeit noch keine ausreichenden Daten vor. Nachteile der Kortikosteroide sind Nebenwirkungen wie Gewichtszunahme, Osteoporose, Verhaltensauffälligkeiten, vermindertes Längenwachstum sowie die Gefahr einer Nebennierenrindeninsuffizienz, langfristig auch kardiovaskuläre Folgeschäden durch eine diabetische Stoffwechsellage. Um eine Addison-Krise zu vermeiden, sollten Glukokortikoide auf keinen Fall abrupt abgesetzt werden. Das Auftreten eines Katarakts unter Glukokortikoidtherapie stellt keine Kontraindikation zur Fortsetzung der Therapie dar. Hierbei sollte eine Rücksprache mit dem Augenarzt erfolgen. Das Risiko einer relevanten Gewichtszunahme scheint für Deflazacort geringer zu sein als für Prednison [16]. Sinnvoll ist auch die Ausstellung eines Notfallausweises für die Patienten.

### Ataluren

Mit Ataluren steht seit 2014 eine mutationsspezifische, dystrophinwiederherstellende Substanz für die DMD-Therapie zur Verfügung. Ataluren kann bei Patienten eingesetzt werden, deren Erkrankung auf einer Nonsense-Mutation im Dystrophingen (nmDMD) mit einem vorzeitigen Stopp-Codon auf mRNA-Ebene beruht [4, 17] – das sind ca. 13 % aller DMD-Patienten (www.dmd-register.de). Ataluren fügt eine Aminosäure anstelle des Nonsense-Codons ein. Dies bewirkt ein ribosomales „Überlesen“ („read-through“) des vorzeitigen Stopp-Codons und damit die Bildung größerer Mengen funktionell aktiven Dystrophins [12, 17]. In placebokontrollierten, randomisierten Studien der Phasen IIb (*n* = 174) und III (DMD-ACT; *n* = 230) konnten gehfähige nmDMD-Patienten unter einer Therapie mit Ataluren 40 mg/kg/Tag motorische und körperliche Funktionen über 48 Wochen im Vergleich zu Placebo länger aufrechterhalten [6, 17, 20, 23]. Zwar verbesserte sich der 6‑Minuten-Gehtest (6MWT), der das primäre Zielkriterium war, in der gesamten Gruppe nicht signifikant gegenüber Placebo, aber die Patienten an der Grenze zum Verlust der Gehfähigkeit konnten ihre Gehfähigkeit über einen längeren Zeitraum erhalten. Die Reduktion des 6MWT fiel in dieser Subgruppe signifikant geringer aus. In der Bewertung des Gemeinsamen Bundesausschusses (GBA) vom 01.12.2016 wurde zunächst kein Zusatznutzen bei nichtgehfähigen Patienten gesehen (https://www.g-ba.de/bewertungsverfahren/nutzenbewertung/239/), neue Daten legen das aber nahe. So zeigte die Gabe von Ataluren zusätzlich zur Standardtherapie im Krankheitsverlauf einen signifikant verzögerten Verlust der Gehfähigkeit und einen längeren Erhalt proximaler Muskelfunktionen als mit der Standardtherapie alleine [27]. Hierbei wurden anhand einer Propensity-matching-Analyse mit Ataluren behandelte Patienten aus dem STRIDE-Register (*n* = 181, davon 17 nichtgehfähig) mit Patienten der Natural History Study der Cooperative International Neuromuscular Research Group (CINRG DNHS; *n* = 181) verglichen. Bei Ataluren-behandelten Patienten traten zudem die für nichtgehfähige Patienten relevanten Meilensteine einer pulmonalen Verschlechterung (FVC%p <60 %, FVC%p <50 %, FVC <1 l) in einem höheren Lebensalter auf als bei gematchten Patienten der CINRG-DHNS-Kohorte. In allen Studien waren Patienten sowohl mit als auch ohne gleichzeitige Therapie mit Glukokortikoiden eingeschlossen. Ataluren zeigte eine gute Verträglichkeit, unerwünschte Ereignisse traten mit Ausnahme von Diarrhö, Erbrechen und Infektionen der oberen Atemwege etwa gleich häufig in der Behandlungsgruppe wie unter Placebo auf.

Die Therapie mit Ataluren ist bei Patienten, deren DMD auf einer Nonsense-Mutation beruht, ab einem Alter von 2 Jahren zugelassen [3]. Ataluren kann Patienten mit und ohne gleichzeitige Glukokortikoidtherapie verabreicht werden. Hinweise für eine verzögerte Krankheitsprogression im Hinblick auf relevante pulmonologische Endpunkte sprechen dafür, die Therapie mit Ataluren auch nach Verlust der Gehfähigkeit als „off-label use“ fortzuführen.

### Eteplirsen, Golodirsen

Die Antisense-Oligonukleotide Eteplirsen und Golodirsen zählen wie Ataluren zu den mutationsspezifisch wirksamen Substanzen. Durch Entfernung von Exon 51 (Eteplirsen) bzw. Exon 53 (Golodirsen) beim Splicing der Dystrophin-prä-mRNA (Exon-Skipping) wird die Expression des Dystrophinproteins wieder hergestellt [37, 38].

Bei mit Eteplirsen behandelten DMD-Patienten wurde in einer kleinen doppelblinden, placebokontrollierten randomisierten Studie (*n* = 8) ein Anstieg von Dystrophinprotein in der Skelettmuskulatur beobachtet [9, 26]. In einer randomisierten Studie zeigte sich bei mit Eteplirsen in einer Dosierung von 30 oder 50 mg/kg (*n* = 12, davon 2 nichtgehfähige Patienten) behandelten Kindern eine verzögerte Abnahme der 6MWD über 24 Wochen im Vergleich zu gematchten Patienten einer historischen Kohorte (*n* = 13; [1, 25]). Dieser Effekt hielt in einer Fortsetzungsstudie über 3 Jahre an; eine Zulassung der FDA erfolgte allerdings nicht vor dem Hintergrund funktioneller Besserung, sondern aufgrund einer gesteigerten Dystrophinexpression. Die pulmonale Situation der Patienten blieb stabil. Golodirsen induzierte bei Patienten, die für ein Exon-53-Skipping infrage kommen, die Bildung von Dystrophin in der Skelettmuskulatur [13, 29]. Zur Behandlung erwachsener Patienten mit einer Exon-Skipping-Therapie liegen derzeit noch keine belastbaren Daten vor. Eteplirsen und Golodirsen sind in den USA für die mutationsspezifische Therapie von Patienten mit den jeweils passenden Deletionen im DMD-Gen zugelassen. In Europa ist dies bisher nicht der Fall.

### Idebenon

Einen anderen Wirkansatz weist Idebenon als synthetisches Analogon des Antioxidans Koenzym Q10 auf. Der Wirkmechanismus ist nicht vollständig geklärt. Vermutlich kann die Substanz Elektronen direkt an den Komplex III der mitochondrialen Atmungskette übertragen, somit den Komplex I umgehen und die Gewinnung von ATP bei Komplex-I-Defekt wieder herstellen [7].

Für Idebenon in einer Dosierung von 900 mg/Tag konnte in der Phase-III-Studie DELOS über 52 Wochen bei jugendlichen DMD-Patienten (*n* = 64), die keine Glukokortikoide erhielten, eine geringer ausgeprägte Verschlechterung der Lungenfunktion – gemessen als PEF, PEF%p, FVC und FEV_1_ – im Vergleich zu Placebo dokumentiert werden [8, 22]. Gleichzeitig war eine signifikante Reduktion respiratorischer Infekte zu beobachten [24]. Diese Ergebnisse wurden seitens der EMA allerdings für eine Zulassung als noch nicht ausreichend angesehen. Die retrospektive Kohortenstudie SYROS mit Patienten der DELOS-Studie (*n* = 18) bestätigte eine signifikant geringere Abnahme der Lungenfunktion im Vergleich zu unbehandelten Patienten der CINRG-DNHS-Kohorte in der Langzeittherapie [34]. Die Verträglichkeit von Idebenon war bis auf eine häufigere Diarrhö mit der von Placebo vergleichbar [8].

Eine Wirksamkeit von Idebenon bei gleichzeitiger Steroidtherapie ist aktuell nicht nachgewiesen, wird allerdings aktuell in einer klinischen Studie (SIDEROS) überprüft [10]. Zur Wirksamkeit im Hinblick auf Muskelkraft und motorische Funktionen liegen für Idebenon keine Daten vor. Bisher ist Idebenon nur für die Behandlung der Leberschen hereditären Optikusneuropathie, aber nicht für Patienten mit DMD zugelassen.

## Auswahl der medikamentösen Zusatztherapie

### Konsensus

Patienten mit DMD sollte neben supportiven und symptomatischen Maßnahmen zusätzlich eine medikamentöse Therapie angeboten werden, die den Verlauf der Erkrankung abmildern oder modifizieren kann. Vor dem Hintergrund der limitierten Behandlungsoptionen, der klinischen Erfahrung und pathogenetisch begründeten Ansätzen kann die Fortführung der Therapie der Betroffenen nach Verlust der Gehfähigkeit gerechtfertigt sein.

- Glukokortikoide stellen eine medikamentöse Standardtherapie bei DMD dar. Die Therapie kann nach Verlust der Gehfähigkeit in reduzierter Dosierung bei entsprechender Verträglichkeit fortgeführt werden. Bei relevanter Gewichtszunahme unter Prednisolon ist ggf. Deflazacort als Alternative in Betracht zu ziehen.
- Für Patienten mit DMD aufgrund einer Nonsense-Mutation steht Ataluren als krankheitsmodifizierende, dystrophinwiederherstellende Therapie zur Verfügung. Hinweise auf eine verzögerte Krankheitsprogression bez. des Zeitpunkts des Verlusts der Gehfähigkeit sowie der Verschlechterung der proximalen Muskelkraft und der Lungenfunktion sprechen für eine Fortsetzung der Gabe von Ataluren auch bei nichtgehfähigen Patienten.
- Das Konzept des Exon-Skipping ist ein interessanter molekularer Therapieansatz. Derzeit liegen jedoch noch keine belastbaren Daten mit größeren Studienkollektiven vor, die den Einsatz des Exon-Skipping bei erwachsenen Patienten rechtfertigen würden.
- Idebenon verfügt als Antioxidans über einen anderen Wirkmechanismus als mutationsspezifische Substanzen. Die Wirksamkeit auf pulmonale Endpunkte ist bei jugendlichen Patienten ohne gleichzeitige Glukokortikoidtherapie dokumentiert. Ein Off-label-Einsatz von Idebenon ist derzeit am ehesten bei nichtgehfähigen, jugendlichen Patienten in Betracht zu ziehen, für die es keine therapeutische Alternative gibt und die nicht mit Glukokortikoiden behandelt werden können.
- Bezüglich der kombinierten Anwendung kann die Behandlung mit Ataluren unabhängig davon erfolgen, ob die Patienten Glukokortikoide erhalten oder nicht. Die Kombination von Idebenon und Glukokortikoiden wird derzeit in einer klinischen Phase-III-Studie (SIDEROS) überprüft [10]. Bei nichtgehfähigen nmDMD-Patienten könnte eine Add-on-Therapie mit Idebenon zusätzlich zu Ataluren in Betracht gezogen werden. Derzeit liegen für diesen Ansatz allerdings keine Daten vor

## Fazit

- Für alle Patienten mit DMD und ihre Familien ist ein interdisziplinärer, integrativer Therapieansatz über die gesamte Lebensspanne und über Schlüsselereignisse wie den Verlust der Gehfähigkeit oder den Wechsel der Betreuung von der pädiatrischen Versorgung in die Erwachsenenmedizin hinaus von zentraler Bedeutung.
- Medikamentöse Maßnahmen, die den Krankheitsverlauf auch nach Verlust der Gehfähigkeit positiv beeinflussen können, umfassen zunächst die Glukokortikoidtherapie, die Fortsetzung der Therapie mit Ataluren bei Patienten mit nmDMD und ggf. eine Off-label-add-on-Gabe von Idebenon, sofern keine Glukokortikoide gegeben werden und es keine therapeutische Alternative gibt.
- Interdisziplinäre symptomatische Therapien und die Anbindung an neuromuskuläre Zentren sind auch nach Verlust der Gehfähigkeit von entscheidender Bedeutung zur Vermeidung von Komplikationen und Verbesserung der Lebensqualität.
- „Bislang liegen keine nationalen oder internationalen Leitlinien zur Behandlung von DMD vor, auch in publizierten Konsensusempfehlungen [2, 3] sind medikamentöse Therapien – abgesehen von der nicht allgemein gehandhabten Empfehlung, Kortikosteroide nach Verlust der Gehfähigkeit nicht zu beenden – bei nichtgehfähigen Patienten bisher nicht berücksichtigt.“
- Fortschritte in der Therapie von Patienten mit DMD sind durch neue molekulare und genetische Therapieansätze zukünftig zu erwarten [39].

Für diesen Beitrag wurden von den Autoren keine Studien an Menschen oder Tieren durchgeführt. Für die aufgeführten Studien gelten die jeweils dort angegebenen ethischen Richtlinien.

## References

[1] Alfano LN, Charleston JS, Connolly AM (2019). Long-term treatment with eteplirsen in nonambulatory patients with Duchenne muscular dystrophy. Medicine.

[2] Birnkrant DJ, Bushby K, Bann CM (2018). Diagnosis and management of Duchenne muscular dystrophy, part 2: respiratory, cardiac, bone health, and orthopaedic management. Lancet Neurol.

[3] Birnkrant DJ, Bushby K, Bann CM (2018). Diagnosis and management of Duchenne muscular dystrophy, part 1: diagnosis, and neuromuscular, rehabilitation, endocrine, and gastrointestinal and nutritional management. Lancet Neurol.

[4] Bladen CL, Salgado D, Monges S (2015). The TREAT-NMD DMD global database: analysis of more than 7,000 Duchenne muscular dystrophy mutations. Hum Mutat.

[5] Bushby K, Finkel R, Birnkrant DJ (2010). Diagnosis and management of Duchenne muscular dystrophy, part 1: diagnosis, and pharmacological and psychosocial management. Lancet Neurol.

[6] Bushby K, Finkel R, Wong B (2014). Ataluren treatment of patients with nonsense mutation dystrophinopathy. Muscle Nerve.

[7] Buyse GM, Goemans N, van den Hauwe M (2011). Idebenone as a novel, therapeutic approach for Duchenne muscular dystrophy: results from a 12 month, double-blind, randomized placebo-controlled trial. Neuromuscul Disord.

[8] Buyse GM, Voit T, Schara U (2015). Efficacy of idebenone on respiratory function in patients with Duchenne muscular dystrophy not using glucocorticoids (DELOS): a double-blind randomised placebo-controlled phase 3 trial. Lancet.

[9] Charleston JS, Schnell FJ, Dworzak J (2018). Eteplirsen treatment for Duchenne muscular dystrophy: Exon skipping and dystrophin production. Neurology.

[10] ClinicalTrials.gov (2018). Phase III study with Idebenone in patients with Duchenne muscular dystrophy (DMD) taking glucocorticoid steroids (SIDEROS).

[11] Connolly AM, Malkus EC, Mendell JR (2015). Outcome reliability in non-ambulatory boys/men with Duchenne muscular dystrophy. Muscle Nerve.

[12] Finkel RS, Flanigan KM, Wong B (2013). Phase 2a study of ataluren-mediated dystrophin production in patients with nonsense mutation Duchenne muscular dystrophy. PLoS ONE.

[13] Frank DE, Schnell FJ, Akana C (2020). Increased dystrophin production with golodirsen in patients with Duchenne muscular dystrophy. Neurology.

[14] Garcia S, de Haro T, Zafra-Ceres M (2014). Identification of de novo mutations of Duchenne/Becker muscular dystrophies in southern Spain. Int J Med Sci.

[15] Goemans N, van den Hauwe M, Wilson R (2013). Ambulatory capacity and disease progression as measured by the 6-minute-walk-distance in Duchenne muscular dystrophy subjects on daily corticosteroids. Neuromuscul Disord.

[16] Griggs RC, Miller JP, Greenberg CR (2016). Efficacy and safety of deflazacort vs prednisone and placebo for Duchenne muscular dystrophy. Neurology.

[17] Haas M, Vlcek V, Balabanov P (2015). European medicines agency review of ataluren for the treatment of ambulant patients aged 5 years and older with Duchenne muscular dystrophy resulting from a nonsense mutation in the dystrophin gene. Neuromuscul Disord.

[18] Humbertclaude V, Hamroun D, Bezzou K (2012). Motor and respiratory heterogeneity in Duchenne patients: implication for clinical trials. Eur J Paediatr Neurol.

[19] Khan N, Eliopoulos H, Han L (2019). Eteplirsen treatment attenuates respiratory decline in ambulatory and non-ambulatory patients with Duchenne muscular dystrophy. J Neuromuscul Dis.

[20] Li D, McDonald CM, Elfring GL (2020). Assessment of treatment effect with multiple outcomes in 2 clinical trials of patients with Duchenne muscular dystrophy. JAMA Netw Open.

[21] Matthews E, Brassington R, Kuntzer T (2016). Corticosteroids for the treatment of Duchenne muscular dystrophy. Cochrane Database Syst Rev.

[22] Mayer OH, Leinonen M, Rummey C (2017). Efficacy of idebenone to preserve respiratory function above clinically meaningful thresholds for forced vital capacity (FVC) in patients with Duchenne muscular dystrophy. J Neuromuscul Dis.

[23] Mcdonald CM, Campbell C, Torricelli RE (2017). Ataluren in patients with nonsense mutation Duchenne muscular dystrophy (ACT DMD): a multicentre, randomised, double-blind, placebo-controlled, phase 3 trial. Lancet.

[24] McDonald CM, Meier T, Voit T (2016). Idebenone reduces respiratory complications in patients with Duchenne muscular dystrophy. Neuromuscul Disord.

[25] Mendell JR, Goemans N, Lowes LP (2016). Longitudinal effect of eteplirsen versus historical control on ambulation in Duchenne muscular dystrophy. Ann Neurol.

[26] Mendell JR, Rodino-Klapac LR, Sahenk Z (2013). Eteplirsen for the treatment of Duchenne muscular dystrophy. Ann Neurol.

[27] Mercuri E, Muntoni F, Osorio AN (2020). Safety and effectiveness of ataluren: comparison of results from the STRIDE registry and CINRG DMD natural history study. J Comp Eff Res.

[28] Mercuri E, Signorovitch JE, Swallow E (2016). Categorizing natural history trajectories of ambulatory function measured by the 6-minute walk distance in patients with Duchenne muscular dystrophy. Neuromuscul Disord.

[29] Muntoni F, Frank D, Sardone V (2018). Golodirsen induces Exon skipping leading to sarcolemmal dystrophin expression in Duchenne muscular dystrophy patients with mutations amenable to Exon 53 skipping (S22.001). Neurology.

[30] Nowak KJ, Davies KE (2004). Duchenne muscular dystrophy and dystrophin: pathogenesis and opportunities for treatment. EMBO Rep.

[31] Paramsothy P, Herron AR, Lamb MM (2018). Health care transition experiences of males with childhood-onset Duchenne and Becker muscular dystrophy: findings from the muscular dystrophy surveillance tracking and research network (MD STARnet) health care transitions and other life experiences survey. PLoS Curr.

[32] Ricotti V, Selby V, Ridout D (2019). Respiratory and upper limb function as outcome measures in ambulant and non-ambulant subjects with Duchenne muscular dystrophy: a prospective multicentre study. Neuromuscul Disord.

[33] Ryder S, Leadley RM, Armstrong N (2017). The burden, epidemiology, costs and treatment for Duchenne muscular dystrophy: an evidence review. Orphanet J Rare Dis.

[34] Servais L, Straathof CSM, Schara U (2020). Long-term data with idebenone on respiratory function outcomes in patients with Duchenne muscular dystrophy. Neuromuscul Disord.

[35] Spurney C, Shimizu R, Morgenroth LP (2014). Cooperative international neuromuscular research group Duchenne natural history study demonstrates insufficient diagnosis and treatment of cardiomyopathy in Duchenne muscular dystrophy. Muscle Nerve.

[36] Steffensen BF, Lyager S, Werge B (2002). Physical capacity in non-ambulatory people with Duchenne muscular dystrophy or spinal muscular atrophy: a longitudinal study. Dev Med Child Neurol.

[37] van Deutekom JC, Janson AA, Ginjaar IB (2007). Local dystrophin restoration with antisense oligonucleotide PRO051. N Engl J Med.

[38] Verhaart IE, Aartsma-Rus A (2012). Gene therapy for Duchenne muscular dystrophy. Curr Opin Neurol.

[39] Verhaart IEC, Aartsma-Rus A (2019). Therapeutic developments for Duchenne muscular dystrophy. Nat Rev Neurol.

[40] Vry J, Schara U, Lutz S (2012). Diagnose und Therapie der Muskeldystrophie Duchenne. Monatsschr Kinderheilkd.

